# Combined spectroscopic and microscopic investigation of stability upon atmospheric exposure of Ag nanoparticle solutions produced by sputtering onto rapeseed oil

**DOI:** 10.1039/d5na01153a

**Published:** 2026-03-19

**Authors:** Héloïse Lasfargues, Devi Janani Ramesh, Lilli Charlotte Freymann, Jochen M. Schneider, Clio Azina

**Affiliations:** a Materials Chemistry, RWTH Aachen University Kopernikusstraße 10 52074 Aachen Germany lasfargues@mch.rwth-aachen.de

## Abstract

Ag nanoparticle (NP) solutions were produced by magnetron sputtering onto rapeseed oil and their stability under atmospheric exposure up to 35 days was investigated by infrared (IR), ultraviolet-visible (UV-vis), X-ray photoelectron spectroscopy (XPS), and electron microscopy. The as-synthesized solutions were dark brown, due to the localized surface plasmon resonance (LSPR) of the Ag NPs. Only ∼10% of all NPs were > 2 nm in diameter, with a median size of 1.4 (−0.3/+0.4) nm. Besides Ag NPs, Ag–AgCl Janus-type NPs were detected, whose formation was attributed to the presence of Cl containing triglycerides in the oil. Within 35 days, the color of the solutions gradually changed to light orange and the proportion of NPs > 2 nm increased by almost 20%. The redshift of ∼15 nm and absorbance decay of the LSPR on the UV-vis spectra after 35 days were mainly attributed to the continued formation of Ag–AgCl NPs and the presence of Ag compounds, like Ag–S–O. Furthermore, oil oxidation, revealed, in IR, by the formation of hydroperoxides upon atmospheric exposure, was promoted by the presence of Ag NPs. Finally, XPS investigations on the solutions revealed the interaction, at all exposure times, between carboxylate groups (–COO^−^) and Ag, suggesting the opening of triglycerides and/or deprotonation of fatty acids. Besides shedding light experimentally on the interaction between NPs and liquid, this work shows the mutual influence of NP and liquid on each other, and underlines the crucial role of trace contaminants in the host liquid on NP chemistry.

## Abstract 2

Oil molecules interact *via* the carboxylate group of their fatty acids with Ag NPs produced by sputtering onto rapeseed oil.

## Graphical abstract

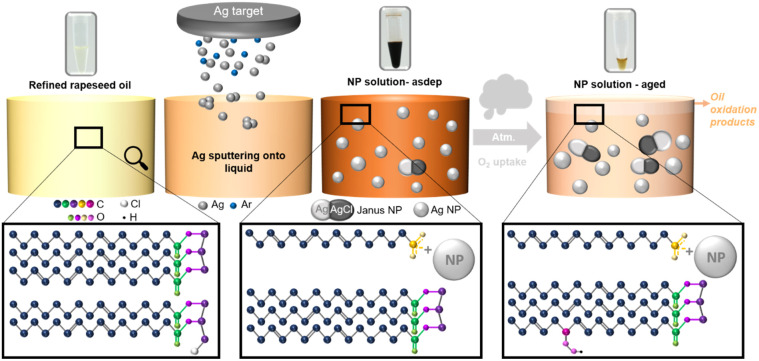

## 1. Introduction

Sputtering onto liquids (SoL) allows the production of colloidal nanoparticles (NPs) in a single step process requiring only a target, as NP material source, and a host liquid. The purity of the NPs produced by SoL is a great advantage of this technique compared to the wet chemistry processes, since it is often required for various applications, *e.g.* in catalysis.^1^ Beyond purity and NP properties like size and shape, stability over time is necessary to enable practical use and storage.

While sputtering parameters have been shown to influence the stability of solutions processed by SoL,^2^ the latter is primarily determined by the combination of NP material and host liquid.^3^ While a plethora of NP materials are conceivable, the choice of the liquid is restricted by its vapor pressure. Ionic liquids offer a vast variety of vacuum-sustaining fluids, but their toxicity can be an obstacle to their practical use. Alternatives can be found among biocompatible and biodegradable materials, like vegetable oils, which have been used successfully in SoL,^4,5^ and were chosen for the present investigation.

To describe solution stability, the evolution of NP size with time can be assessed by imaging techniques such as transmission electron microscopy (TEM), which is widely used in SoL studies.^6–8^ For plasmonic NPs, like gold (Au), silver (Ag) or copper (Cu), the solution stability over time can easily be monitored using UV-vis spectroscopy,^9^ thereby complementing TEM investigations.^6–8^ Indeed, such NPs exhibit a localized surface plasmon resonance (LSPR) due to the collective oscillation of conduction electrons upon interaction with incoming electromagnetic waves at specific wavelengths.^10^ The position, shape and intensity of the LSPR results from NP and medium refractive index,^11^ as well as NP crystallographic structure,^12^ shape,^13^ and size distribution.^11^ However, the sole monitoring of solution stability over time does not allow a thorough understanding of it and associated stabilization mechanism. Due to the large surface to volume ratio of NPs, attractive van der Waals forces between particles tend to destabilize colloidal solutions,^14^ therefore, sufficiently strong repulsive forces between NPs are crucial for stability. The main repulsive forces found in such systems are electrical forces, due to charges on the surface of the particles, and steric forces, in case molecules like polymers or surfactants adsorb on the particle surface.^14^ In mostly non-polar media like vegetable oils, electrical forces are not likely to dominate; without addition of further chemicals, the liquid therefore needs to act as a steric stabilizer to ensure colloidal stability for the chosen NP material. Hence, describing the system-specific interaction between NPs and host liquid molecules is key to understanding NP and solution stability. While this interaction is well reported for surfactants used in wet chemistry approaches,^15^ it is not yet fully understood in the case of colloids obtained by SoL.

Reports have used density functional theory (DFT) calculations to assess whether the interaction between a model molecule and a specific surface orientation of a NP is energetically favorable^2,16–21^ and could therefore be expected to balance out attractive forces between NPs. For instance, Sergievskaya *et al.* calculated the interaction energy between a third of a triglyceride of ricinoleic acid and a Au or Ag (111) surface and found that the interaction is favorable only in the case of Au.^2,17^ However, the necessity to model only part of the liquid molecule and to choose specific functional groups to probe the interaction are limitations to these theoretical approaches. While the reported calculation results allowed authors to explain differences in solution stability between *e.g.* two NP materials, no experimental proof of the proposed mechanisms were reported. In other studies, the changes observed in NP size and solution stability, for example, between different host liquids were explained based on expected interactions between the NP material and the liquid molecule, making use of knowledge from wet chemistry synthesis.^22–25^ For instance, Porta *et al.* and Ishida *et al.* controlled Cu and Ag NPs sizes upon addition of 11-mercaptoundecanoic acid (MUA) to polyethylene glycol (PEG),^23,24^ the former being a common capping agent for metallic NPs in wet chemistry synthesis.

Experimentally, organic-metal interfaces can be probed and described by X-ray photoelectron spectroscopy (XPS). For instance, the reactivity of different metals sputtered onto a polymer surface were compared in terms of relative interfacial bond concentration determined by XPS measurements, in the frame of adhesion studies.^26^ Other studies dealt with the interaction between metal and organic self-assembled monolayers (SAMs) with different functional groups.^27–29^ In the liquid phase, XPS measurements on colloids have been performed using a liquid microjet, to probe the interface between volatile liquids such as water, and NPs,^30^ or by environmental XPS to assess the molecular arrangement around NPs.^16,31^ In the latter case, Biliak *et al.*, did so for Cu NPs produced by sputtering onto PEG from a gas aggregation source.^16,31^

While the research on SoL has significantly increased over the last two decades,^32^ comprehensive studies are still required, considering the great variety of materials combinations and the complexity of the technique, at the crossroads between plasma-based synthesis and colloidal synthesis. Herein, we use a combination of spectroscopic techniques to thoroughly describe the stability of Ag NPs produced by sputtering onto refined rapeseed oil and probe the NP-oil interface to gain insights on the stabilization mechanisms at the molecular level. The colloids were characterized by IR spectroscopy, UV-vis spectroscopy and TEM to track the solution ageing up to 35 days, in order to describe the stability of the host liquid, NPs size evolution, and chemical composition. We assess the interaction between oil molecules and NPs by XPS measurements directly on the solution. To our knowledge, this method is used for the first time in the frame of sputtering onto non-ionic liquids. This study thereby reports on a comprehensive methodology to describe colloids and investigate stability in the frame of SoL and contributes towards a more knowledge-based material choice.

## 2. Experimental

### 2.1. Sputtering onto liquids

Refined rapeseed oil (Oleum Rapae raffinatum; CAS number 93165-31-2; Caesar & Lorentz GmbH) was used as host liquid. The oil was stored in a fridge to minimize ageing and degassed prior to sputtering, using the following procedure. About 3 mL of liquid were degassed in a glass vial for at least 2 days in the load-lock of the sputtering chamber (pressure below 7 × 10^−5^ Pa). A volume of 300 µL of degassed oil were subsequently transferred to a custom-designed aluminium substrate holder, consisting of 4 containers of 16 mm in diameter and 5 mm in height (see Fig. S1 in the SI). The mass of oil used was measured with an analytical weighing scale M-Power AZ64 (Sartorius AG, Göttingen, Germany), with a readability of 0.1 mg. Additionally, a similar volume of oil (∼300 µL) was poured in a glass vial of ∼16 mm in diameter, to be used as reference for comparison with the Ag NP solution. The substrate holder and the glass vial were placed in the load-lock for further degassing of the liquid for at least 3 days. At the end of the procedure, a pressure below 5.5 × 10^−6^ Pa was reached in the load-lock and the holder was transferred to the main chamber. A custom-designed laboratory-scale vacuum chamber (base pressure below 9.0 × 10^−5^ Pa) was used to perform the SoL process, using an elemental Ag target (99.99%, m&k GmbH, Kahla, Germany) of 50 mm in diameter. All experiments were run with a DC discharge in constant power mode, at 80 W (RPG-50, MKS Instruments), and at a pressure of 0.7 Pa, in the presence of Ar (99.999%). The distance between the target surface and the liquid surface was 60 mm. A deposition time of about 55 s allowed to reach a final Ag concentration of 1.10 ± 0.15 mg mL^−1^.

### 2.2. Flux measurements

The flux of species arriving at the liquid surface was estimated from thickness measurements of solid Ag films deposited on Si(100) substrates (Crystal GmbH), which were placed at the same position as the liquid during SoL, using the same sputtering conditions. The solid substrates, with a thickness of 0.5 mm, lay 61 mm away from the target. After 2 min, a Ag film thickness of 318 ± 10 nm was reached, as measured using a Keyence VK-9700 laser scanning microscope (Keyence, Osaka, Japan). To derive the flux from the film thickness, a film density of 10.65 g cm^−3^ was used. This value was determined by X-ray reflectivity as described elsewhere.^3^

### 2.3. Post-sputtering sample-handling and characterization

Following the depositions, the solutions were stirred manually and transferred from the sample holder to PCR tubes for storage. The tubes were kept in ambient conditions (temperature 20–25 °C and humidity 20–30%) and in the dark. The solutions were characterized in the as-deposited state and at different atmospheric exposure times, as described in the following paragraphs. The solutions and pure oil samples were measured as soon as possible after breaking the vacuum and the corresponding timings are reported in Table 1. The Ag NP solutions and pure oil in their initial states are referred to as “asdep” (*i.e.* as-deposited) and “asdeg” (*i.e.* as-degassed), respectively. For each atmospheric exposure time, ∼45 µL Ag NP solution and ∼20 µL pure oil were extracted from the PCR tubes for measurement, after manual stirring, regardless of the characterization method.

**Table 1:** Time between venting after SoL and characterization for all methods employed

	Time after venting corresponding to asdep/asdeg state	Further atmospheric exposure times characterized
ATR-FTIR	∼1–2 h	1, 7, 35 days
UV-vis	∼2–3 h	1, 7, 35 days
XPS	15 min + 2.5 h degassing	1, 7, 35 days
TEM	30 min (grid preparation)	7, 35 days

#### 2.3.1 Fourier Transform infrared (FTIR) spectroscopy

A possible chemical degradation of the liquid matrix upon plasma treatment was assessed by FTIR spectroscopy in Attenuated Total Reflection (ATR) mode, using an IRSpirit spectrophotometer (Shimadzu Co., Kyoto, Japan) equipped with a QATR-S single-reflection ATR measurement attachment. ATR-FTIR measurements were performed on the asdep solutions and the asdeg pure oils, as well as after 1, 7, and 35 days of atmospheric exposure. After background acquisition, ∼3 µL of sample was dropped on the diamond prism and the measurement was performed under an incident angle of 45°. Each spectrum was the average of 32 scans collected in the wavenumber range of 400 to 4000 cm^−1^ with a step size of 1.4 cm^−1^. A Happ–Genzel apodization function was applied for the Fourier Transform integration. After baseline subtraction, the spectra were normalized using the peak at ∼1460 cm^−1^ that did not show significant changes over time, as expected from data published on similar systems.^33^ In order to reveal changes in band position or intensity with atmospheric exposure time, the spectrum of the pure degassed oil was subtracted from the spectra of all pure oil and NP solutions.

#### 2.3.2 Ultraviolet-visible (UV-vis) spectroscopy

The optical response evolution of the solution with atmospheric exposure was assessed by UV-vis spectroscopy, using a Lambda 650 instrument (PerkinElmer U.S. LLC, Shelton, USA) in absorbance mode, equipped with a deuterium and a tungsten lamp, covering the UV and visible light range, and controlled over the software UV WinLab 6.4.0.973/2.02.06. For each measurement, a cuvette was filled with ∼800 µL pure degassed oil and measured as reference (baseline). For asdep samples and the solutions that underwent up to 7 days of atmospheric exposure, ∼5 µL of Ag NP solution were added to each cuvette. After 35 days, a larger Ag NP solution volume was used (∼50–60 µL) to compensate for the much weaker absorbance of the solution. All aliquots were weighed using an analytical scale Mettler AE200 (Mettler-Toledo, Greifensee, Switzerland) with a readability of 0.1 mg, to precisely determine the concentration of sample upon dilution and normalize the absorbance data to the original Ag concentration for all measurements. The cuvettes were shaken until complete homogenization of the solution and measured subsequently. For each sample, 3 cuvettes were measured to ensure sufficient statistics over the wavelength range of 350 to 600 nm, with a step size of 1 nm and a scan speed of 266.75 nm min^−1^. The curves presented are the average of the 3 cuvettes measured. The absorbance values measured were corrected for the dilution of the sample in the cuvette and these corrected values are presented.

#### 2.3.3 Transmission electron microscopy (TEM) and scanning transmission electron microscopy (STEM)

For TEM and STEM observations, a lacey-carbon copper grid (Plano GmbH, Wetzlar, Germany) coated with an ultra-thin carbon layer, with 400 mesh size, was prepared as follows. A volume of ∼20 µL of NP solution was diluted in ∼200 µL acetone in a PCR tube and shaken manually to obtain a homogeneous solution. A second dilution step was performed with ∼30 µL of diluted solution and ∼100 µL of acetone, followed by manual shaking. The copper grid was dipped in the diluted solution for about 45 s, after which two 10 µL drops of solution were casted on the grid. After 5 min, the grid was dipped in approx. 3 mL solvent mixture of 2/3 acetone and 1/3 ethanol for 10–15 minutes, to remove excess oil. This cleaning step was repeated twice for 10–15 min and once for ∼3 h, with clean solvent each time. After the cleaning procedure, the grid was left to dry in air for ∼10 min and stored in the load-lock of the sputtering chamber (pressure below 10^−5^ Pa) until observation.

TEM was performed using a JEOL JEM-F200 operated at 200 kV. High-resolution TEM (HR-TEM) images and selected area diffraction (SAED) patterns were acquired on Gatan OneView Camera. Fast Fourier transforms (FFT) of HR-TEM images were generated using DigitalMicrograph software. STEM images were acquired in dark-field (DF) mode, and energy-dispersive X-ray spectra (EDX) for chemical composition analysis of selected regions were collected using an Oxford AZtecEnergy detector.

To determine NP size distributions, STEM images were taken with a FEI Helios Nanolab 660 dual-beam microscope, with an acceleration voltage of 30 kV, in bright field (BF). For each sample, over 44 images were recorded in total at different magnifications, from at least 3 randomly chosen grid positions. This ensured sufficient measurement statistics (SI, Table S1). Processing and evaluation was carried out using the Fiji software (ImageJ 2.3.0)^34^ and the Particle sizer plugin,^35^ following the methodology described in ref. 3. The NP sizes are given in terms of area equivalent circle diameters of the detected particles.

#### 2.3.4 X-ray photoelectron spectroscopy (XPS)

To characterize the interface between oil and NPs, XPS measurements were carried out on ∼3 µL of pure oil and solution. These volumes were pipetted from the PCR tubes to the cavities (4 mm diameter and 0.35 mm deep) of a custom-designed sample holder (Fig. S3). A total vacuum time of 2.5 h prior to measurement was adopted, comprising the pumping in the load-lock and measurement preparation time in the main chamber. The measurements were carried out in an AXIS SUPRA XPS system (Kratos Analytical Ltd, Manchester, UK), equipped with a monochromatic Al-Kα X-ray source and a hemispherical detector, and controlled over the software ESCApe 1.4.0.1149. The base pressure was below 3 × 10^−6^ Pa. Charging effects were avoided by using a charge neutralizer (low-energy, electron-only source) during the measurements. A survey scan was acquired (80 eV pass energy, step size 0.25 eV, 1 sweep, 80 ms dwell time) for both the pure oil and NP solutions and showed no additional elements than the expected C, O and C, O, Ag, respectively (Fig. S4). High-resolution core-level spectra of C 1s, O 1s, Ag 3d and Auger spectrum Ag MNN were acquired with a pass energy of 40 eV and a step size of 0.075 eV (1 sweep, 500 ms dwell time). These scan parameters led to an energy resolution of ∼0.7 eV, as determined by measuring the full width at half maximum (FWHM) of the Ag 3d 5/2 peak of a sputter-etched Ag foil. For all sample and pure oil drops, the C 1s region was acquired to allow for energy scale calibration, which was performed by setting the peak maximum of the C 1s spectrum to 285 eV, corresponding to the contribution of the C–C and C–H components.^36^ The region Ag MNN was measured on 4 drops (along with Ag 3d), and these spectra were averaged to improve the signal-to-noise ratio, after ensuring no significant changes were observed with vacuum time.

Spectra analysis and quantification were carried out with the CasaXPS software package (Casa Software Ltd+ 2.3.26PR1.0).^37^ For all regions, a Tougaard background was adopted. For quantitative chemical analysis, the areas of all core level spectra and contributions were corrected to account for the analyser transmission function, as well as for the photoelectron escape depth, the photoelectron cross-section, and instrument geometry, using the relative sensitivity factors provided by the manufacturer of the XPS system (Kratos Analytical Ltd). The proportions reported are given with ∼10% accuracy and the peak positions with ± 0.1 eV uncertainty. The C 1s and O 1s spectra on pure oil drops or C 1s, O 1s and Ag 3d spectra on sample drops were used for overall quantification, with proportions of C, O and Ag calculated for each drop measured, and averaged over all drops. The quantification is reported in the SI, Fig. S5. For detailed analysis, peak fitting of the core levels C 1s and O 1s was carried out. The C–C and C–H contribution to the C 1s peak was fitted with a series of 4 mixed Gaussian–Lorentzian (70–30%) vibrational components with decreasing intensities of 40%, 10%, and 5% with respect to the main peak and a constant energy separation of 0.41 eV.^36,38^ For all other contributions on C 1s and all contributions on O 1s, a symmetric mixed Gaussian–Lorentzian (70–30%) peak shape was used. Further details on the measurement and fitting strategy are provided in the SI.

## 3. Results and discussion

**Fig. 1:**
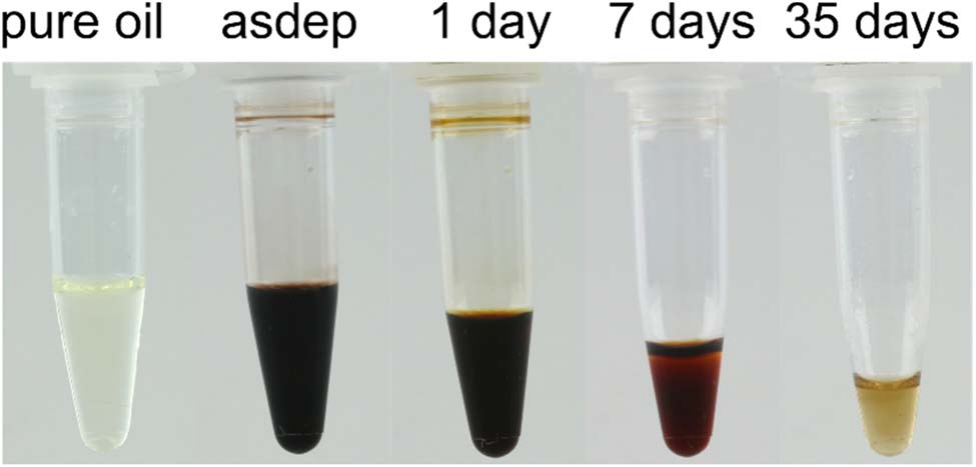
Photographs of the pure rapeseed oil and Ag NP solutions upon atmospheric exposure. The decrease in volume is due to withdrawal of solution for characterization.

Upon Ag sputtering, the clear oil turned dark brown, indicating the presence of Ag-based NPs in high concentration in the asdep sample (Fig. 1). During atmospheric exposure up to 35 days, the Ag NP solutions underwent a colour change from dark brown to light orange. Furthermore, a rancid smell developed with time, which is characteristic of the development of volatile oxidation products in oils.^39^

**Fig. 2:**
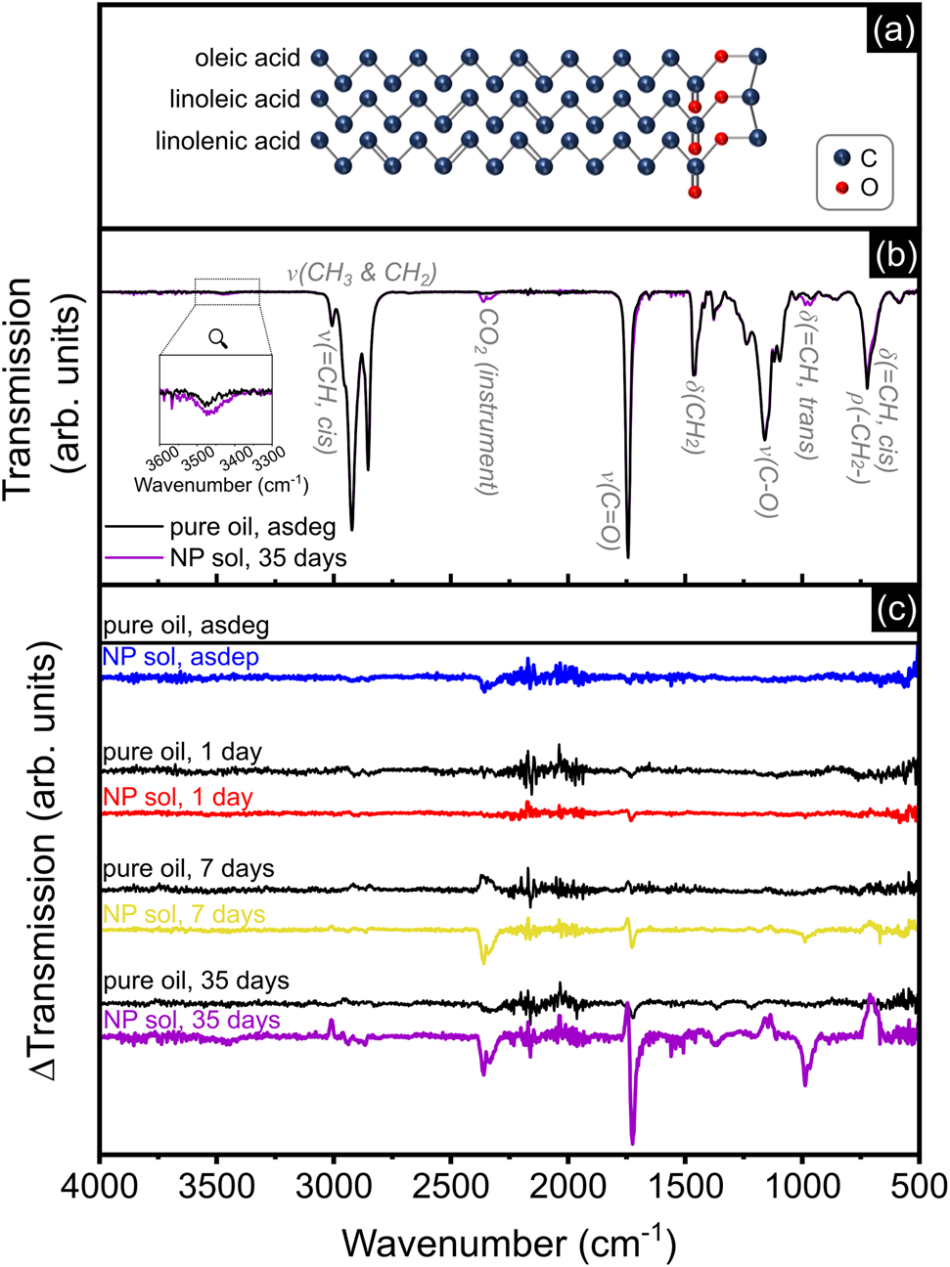
(a) Schematic representation of a triglyceride of rapeseed oil, combining one fatty acid of each of the three main components of the oil. (b) IR transmission spectrum of the pure degassed oil and NP solution at 35 days. (c) Difference in IR transmission of the pure oil (in black) and Ag NP solutions signals in the asdeg and asdep state, respectively, and upon atmospheric exposure (1, 7, and 35 days), referenced to the pure degassed oil. “*ν*”, “*δ*” and “*ρ*” refer to stretching, bending and rocking vibrations, respectively.

The schematic structure of rapeseed oil, given in Fig. 2(a) shows the different functional groups and molecular bonds, giving rise to the vibrations associated to the main absorption bands visible on the IR transmission spectrum of pure degassed rapeseed oil and the NP solution after 35 days of atmospheric exposure (Fig. 2(b)). The symmetrical and asymmetrical stretching vibrations of C–H bonds in CH_2_ and CH_3_ are visible in the region from 2780 to 2990 cm^−1^. The =C–H stretching vibrations arose at 3007 cm^−1^.^40^ The strong band at 1743 cm^−1^ and the weak signal at about 3475 cm^−1^ correspond to the stretching of C=O in the ester group and its overtone, respectively.^40,41^ The intense signal around 1159 cm^−1^ is associated with the stretching vibrations of ester C–O.^40^ The band at ∼1457 cm^−1^ corresponds to the C–H bending vibrations in CH_2_ and CH_3_, whereas the contribution from –(CH_2_)_*n*_– rocking (for *n* ≥ 3) can be seen at 724 cm^−1^.^40^ At ∼700 cm^−1^, the contribution from the C–H out-of-plane bend of *cis*-olefins^40^ overlaps with the signal at 724 cm^−1^. Finally, the signal visible at ∼2350 cm^−1^ originates from gaseous CO_2_ present in the instrument^42^ and is independent of the sample.

In order to detect changes in band positions and intensities upon atmospheric exposure for the pure oil and Ag NP solutions, the IR transmission spectrum of the pure degassed oil was subtracted from the transmission spectra of all samples, as shown in Fig. 2(c). An increase in band intensity (decrease in transmission) with respect to the pure degassed oil translates into a negative transmission difference (ΔTransmission) signal. For the pure oil signal, no clear evolutions were detected up to 7 days over the spectral range investigated. At day 35, there seems to be a slight change in intensity of the band at ∼1750 cm^−1^. Regarding the Ag NP solutions, no significant differences were visible in the asdep state with the signal of the asdeg pure oil. With increasing atmospheric exposure, the signal of Ag NP solutions showed increasing changes over several spectral ranges, as reported in Table 2, and which can be attributed to molecular modifications due to oil oxidation. In particular, several changes are associated with the formation of hydroperoxides, the main primary oxidation products.^33^ Already from day 1, the signal at ∼1720 cm^−1^ increased, leading to the formation of a shoulder at day 35 on the low wavenumber side of the 1743 cm^−1^ band and can be explained by hydrogen bonding effects in the presence of hydroperoxides and/or alcohols.^43^ The two peaks arising at 986 and 967 cm^−1^, clearly visible at day 35, indicated the change from *cis*- to *trans*- configuration of the olefinic bonds (C=C),^43,44^ also associated with the formation of hydroperoxides. This configurational change also explained the drop in intensity of the bands at ∼700 cm^−1^ and at 3007 cm^−1^, identified at day 7 and 35, respectively. The presence of hydroperoxides was further confirmed by the weak signal at ∼3463 cm^−1^, detected at day 35,^43^ and visible on the inset in Fig. 2(b). Further, a slight decrease in intensity is detected, from day 7 on, in the ranges 690–730 cm^−1^ and 1120–1180 cm^−1^, and confirmed at day 35. Finally, the intensity of the signals at 1743 cm^−1^ and in the range 1140–1160 cm^−1^ dropped at day 7 and decreased further at day 35. This indicates a reduction of the proportion of ester groups in the oil, and is attributed to the formation of secondary oxidation products.^33^ These measurements showed that the oil underwent oxidation upon atmospheric exposure, and that the presence of Ag accelerated the process. Such rapid oxidation of the oil matrix was also reported by Michels *et al.* in the case of Au NPs produced by sputtering onto sunflower oil, which is also mainly composed of unsaturated fatty acids.^45^

**Table 2:** Changes observed on the IR transmission spectra of the Ag NP solutions upon atmospheric exposure and corresponding assignments

Atmospheric exposure time when change first detected	Band position (cm^−1^)	Assignment	Behavior
Day 1	1719	Hydrogen bonding effects in presence of hydroperoxides and/or alcohols^11^	Intensity increase (appearance of a shoulder)
Day 1	986 & 967	=C–H bending of conjugated and isolated trans olefins^43,44^	Intensity increase
Day 7	1743	C=O stretching, ester^40^	Intensity decrease
Day 7	∼700	=C–H out-of-plane bending of *cis*-olefins^40^	Intensity decrease
Day 35	3463	OO–H (peroxides)^43^	Intensity increase & broadening
Day 35	3007	=C–H stretching of *cis*-conjugated olefinic bonds^40^	Intensity decrease
Day 35	1140–1160	C–O stretching, ester^40^	Intensity decrease

**Fig. 3:**
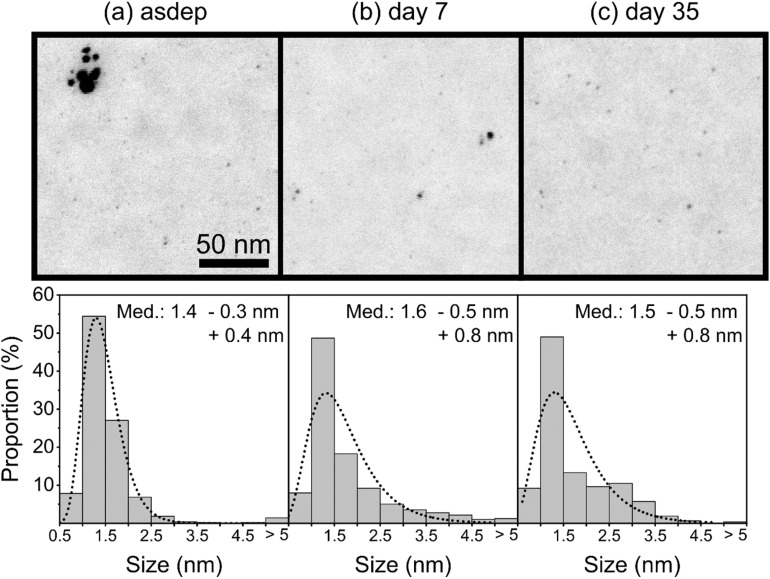
BF STEM images and size distribution of Ag NPs (a) asdep, (b) after 7 days and (c) after 35 days of atmospheric exposure. The dotted lines correspond to the log-normal fit of the size distributions. Median sizes (med.) are reported, along with the distribution width below and above the median.

The size distributions from > 3400 NPs were obtained from STEM images, in the asdep state, and after 7 and 35 days atmospheric exposure time, as shown in Fig. 3(a)–(c), respectively. For NPs up to 5 nm in diameter, representing ∼99% of all NPs measured, the distributions were fitted with a log-normal function to extract the median size (geometric mean, *m*). Using the geometric standard deviation factor (*f*_SD_), the distribution widths above and below the median were determined by computing *m*·*f*_SD_ − *m* and *m* − *m*/*f*_SD_, respectively. For all exposure times, ∼50% of all NPs were 1–1.5 nm in diameter. Main changes occurred between the asdep state and 7 days, with an increase in the proportion of NPs > 2 nm by ∼15%. This suggests that secondary growth processes took place after sputtering and the presence of polycrystalline NPs (SI, Fig. S2) implies that coalescence was part of these processes.

**Fig. 4:**
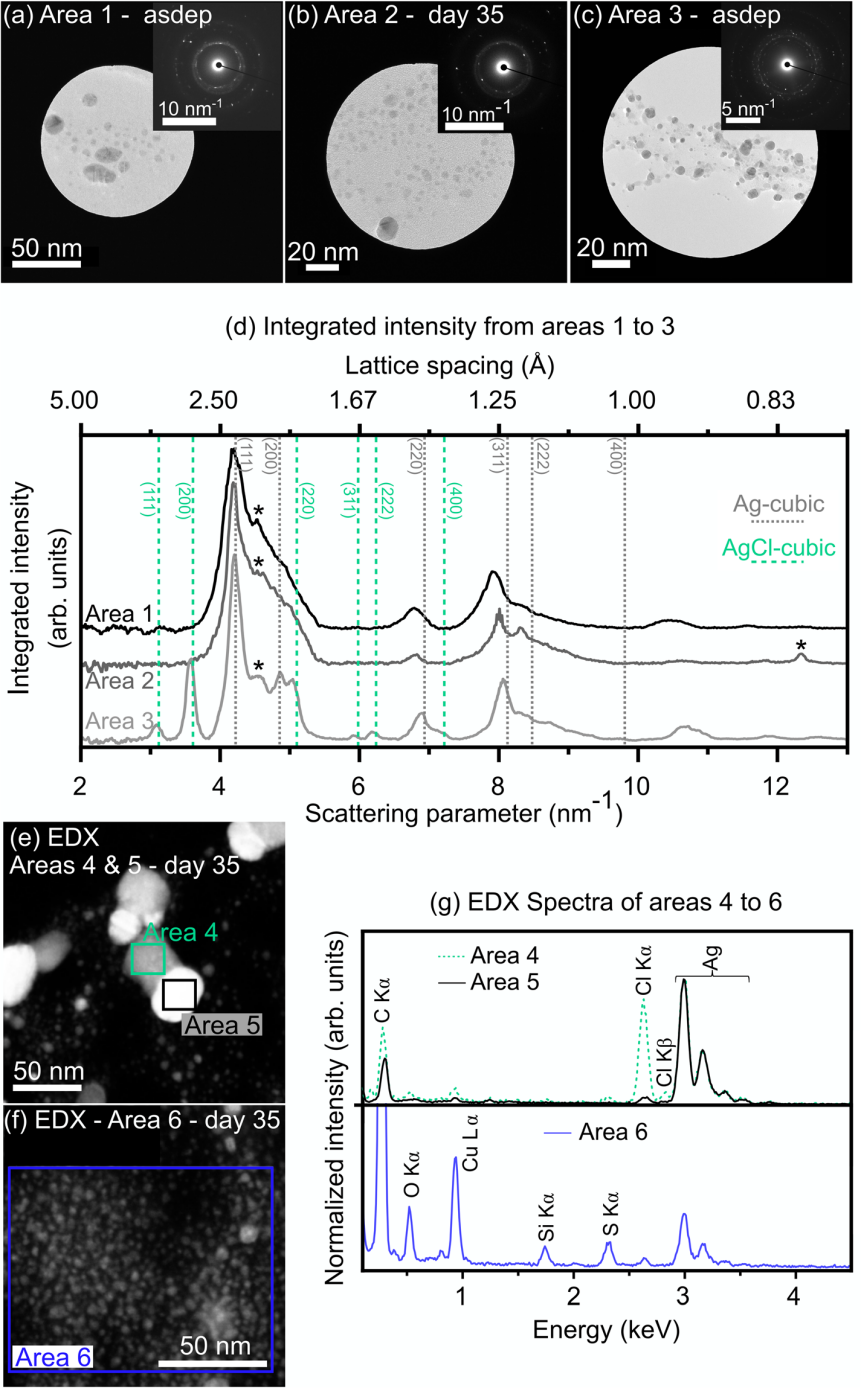
TEM images and corresponding SAED patterns of a population of NP in the asdep state (a and c) and 35 days atmospheric exposure (b), and corresponding integrated intensity (d). DF-STEM images of areas selected for EDX analysis at day 35 (e and f) and corresponding EDX spectra (g). The Si Kα peak (area 6) originates from the STEM detector and not from the sample.

Further investigations were conducted by TEM in order to assess possible changes in the chemistry of the NPs upon atmospheric exposure. SAED patterns with corresponding radial integration profiles are provided in Fig. 4 for asdep and day 35 samples, along with EDX spectra of representative areas for day 35. In all samples investigated, metallic Ag was detected, as shown exemplarily for the asdep state (in Fig. 4(a)) and day 35 (in Fig. 4(b)), from a population of NPs, consisting of mostly small (<5 nm) and a small proportion of large (5–20 nm) NPs. The corresponding radial integration profiles in Fig. 4(d) reveal several peaks in the range 4.2 to 4.9 nm^−1^, as well as around 6.8 and 8.2 nm^−1^. The position of the peaks only partially matched those of face-centered cubic (FCC)–Ag, suggesting that FCC-Ag is not the main contributor to the SAED pattern. While metallic Ag adopts an FCC structure in the bulk state, further size and process-dependent crystal structures have been observed for Ag NPs, such as icosahedra, decahedra or the hexagonal structure.^46–49^ The overall profile shape and peak widths suggest the presence of a large population of icosahedral and decahedral Ag NPs, similarly to the analysis by Reinhart *et al.* on Ag NPs of 2–10 nm in diameter.^48^ In particular, the peak at 4.4 nm^−1^ (asterisk) was shown to indicate the presence of large Ag icosahedra (>5 nm in diameter),^48,50^ which could also explain the peak at ∼12.4 nm^−1^ (asterisk).^48^ The same authors also reported small decahedral Ag particles (∼2–3 nm in diameter) to be responsible for the width of the peaks at ∼7 and 8 nm^−1^.^48^

Along with metallic Ag, AgCl compounds were detected in all samples, as shown for the asdep state in Fig. 4(c) and (d), with peaks at ∼3.1, 3.6, 5 nm^−1^ and around 6 nm^−1^, matching those of (111), (200), (220) and (311) and (222) of cubic AgCl. The presence of Cl in some NPs was also confirmed by EDX analysis (Fig. 4(g), area 4). The formation of AgCl was most probably driven by the presence of Cl contaminations in the refined oil.^51^ Indeed, gas chromatography – tandem mass spectrometry analyses carried out by Eurofins NDSC Food Testing Germany GmbH have shown that the oil contains 340 ± 100 µg kg^−1^ and 180 ± 75 µg kg^−1^ of 2- and 3-monochloropropane-1,2-diol (MCPD), respectively, corresponding to a total of ∼10^15^ Cl atoms per mL oil. The STEM images and EDX analysis showed that the AgCl crystals are found in larger NPs, combined with a metallic Ag domain. Such NPs, with surfaces exhibiting different physical properties, are known as Janus particles. In the present case, the metallic Ag phase appears brighter on the DF-STEM images (area 5 on Fig. 4(e)), while the AgCl part appears darker (area 5 on Fig. 4(e)). Furthermore, the EDX spectrum of area 6 on Fig. 4(g) reveals the presence of O and S in the sample, especially in areas with high densities of small NPs (<10 nm) (Fig. 4(f)). While S can be present as trace contaminant in the oil^52,53^ and while O can arise from oil residues, the incorporation of both elements in the sample could also have occurred during atmospheric exposure of the NP solutions or of the TEM grids. Fast Fourier Transform of HRTEM images suggested that Ag–S–O compounds formed, for instance Ag_2_SO_4_ (see SI, Fig. S2). Although not evidenced by TEM investigations, the presence of Ag oxide phases cannot be ruled out, due to the numerous overlaps on the diffraction patterns of AgCl and the most stable Ag oxide phase, cubic Ag_2_O, to the possible formation of surface suboxides on Ag NPs,^54^ and/or to defects in NPs.^55^

**Fig. 5:**
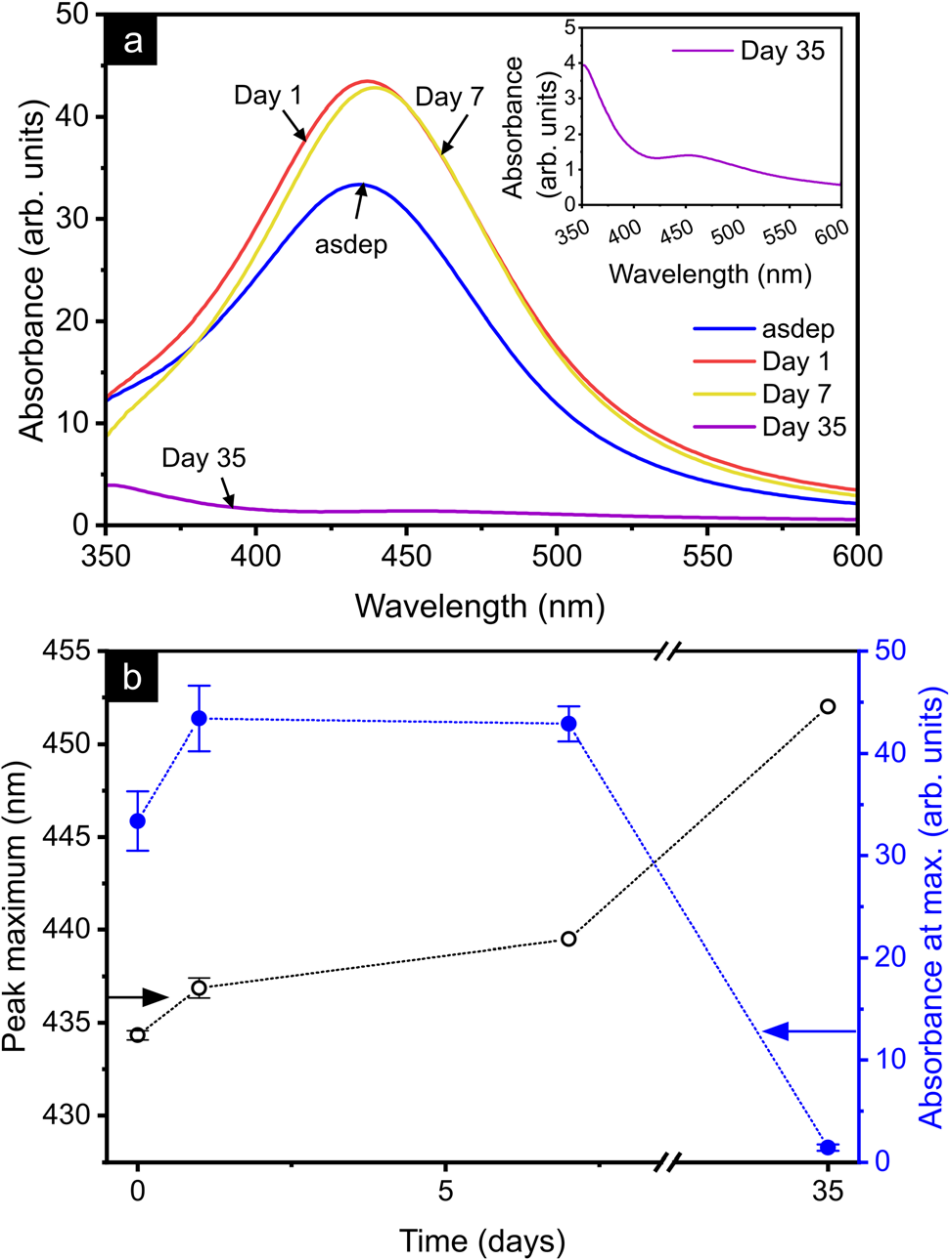
(a) UV-vis spectra of the asdep Ag NP solution and upon atmospheric exposure (1, 7, and 35 days). (b) Evolution of position and intensity of the UV-vis peak maximum with atmospheric exposure. The absorbance was corrected to the original Ag concentration in the solutions.

The stability of the solutions with atmospheric exposure was further monitored by UV-vis spectroscopy, as shown in Fig. 5(a). All spectra exhibited a broad absorption band in the range 430–450 nm, attributed to the LSPR of Ag NPs. The evolution of the position of the peak maximum and maximum absorbance in this range is represented in Fig. 5(b). The error bars given correspond to the standard deviation of the corrected absorbance for the 3 cuvettes prepared for each sample. Larger error bars (not shown here) are obtained when including the uncertainty on the masses of solution and pure oil aliquots used for the cuvette's preparation and on the absorbance value from the instrument, without impacting the trends reported. Up to 7 days of atmospheric exposure, the peak maximum shifted towards larger wavelengths, from 434 nm in the asdep state to 440 nm. This shift was accompanied by an increase in maximum absorbance by ∼30% already at day 1, which remained stable up to day 7. Between day 7 and day 35, the peak position shifted by 15–20 nm to longer wavelengths and the absorbance at the LSPR position dropped by ∼97%. Absorbance signals of NP solutions can be affected by NP sedimentation over time. However, in the present study, aggregation was not observed visually. Moreover, the sample preparation included stirring of the solutions prior to cuvette preparation and shaking of the cuvettes for complete homogenization of the solutions, ensuring redispersion of the NPs in case agglomeration occurred.

Due to the numerous parameters influencing the LSPR (refractive index of NP and medium,^11^ NP crystallographic structure,^12^ shape^13^ and size^11^), the consideration of the UV-vis spectra alone does not allow for an unambiguous attribution of the changes occurring in solution over time. As evidenced by the size distributions, ∼99% of the NPs are < 5 nm in diameter. The LSPR of Ag NPs in the range 2–20 nm in diameter is expected around 410–415 nm and 420 nm, in ethylene glycol^56^ (refractive index of 1.44 at 20 °C (ref. 57)), and glass (refractive index of 1.5),^11^ respectively. Rapeseed oil has a refractive index of 1.47, therefore, the position of the LSPR of 2–20 nm Ag NPs is expected to lie in the range of 410–420 nm. While these reports were based on calculations for spherical Ag NPs, the LSPR of Ag NPs with sizes > 2 nm, produced by SoL in castor oil (refractive index of 1.48 at 27 °C (ref. 58)), was observed in the range 420–435 nm by Sergievskaya *et al.*,^2^ which is in good agreement with the results presented herein. For smaller clusters (<1–1.5 nm in diameter) and atoms, no clear LSPR is expected, but an absorption peak from interband transitions in Ag atoms can arise, located at ∼330 nm for Ag in glass.^59,60^ Due to the strong absorption of the rapeseed oil itself, below 350 nm, such features were not detectable in the system studied here. Therefore, the main UV-vis signal was attributed to Ag NPs. The increase in NP size between the asdep state and day 7 (with ∼15% increase in the proportion of NPs > 2 nm diameter, see Fig. 3) might have led to the observed increase in absorbance of the LSPR between these samples, as expected for NPs up to 20 nm in diameter.^11^ However, the NP size distributions cannot explain the LSPR shift and intensity drop between day 7 and 35, since no significant size change was observed between these times. As for the oil matrix, its strong oxidation after 35 days is expected to only minimally influence the measured UV-vis spectra, as the refractive index was shown by Arya *et al.* to increase by < 0.01 upon oxidation for a series of vegetable oils.^39^ However, the chemical composition of the NPs significantly contributed to the position of the LSPR. As seen in Fig. 4, Janus type Ag–AgCl NPs are present already in the asdep state. Pure AgCl NPs do not exhibit a LSPR on their own;^61^ however, the presence of the AgCl in the Janus NPs investigated is expected to modify the position and intensity of the Ag LSPR. Considering the refractive index of AgCl (1.94–2.47 (ref. 62)), a shift of the LSPR of Ag to higher wavelengths is expected for Ag–AgCl NPs.^63^ Such shifts and absorbance decays could also originate from the formation of further Ag compounds, for instance upon partial oxidation of Ag NPs, as reported for Ag@Ag_2_O (core@shell) NPs.^64,65^ Therefore, the drop in LSPR absorbance and wavelength shift occurring between day 7 and day 35 was associated with chemical changes of the NPs, and suggest the formation of increased proportions of Ag compounds (*e.g.* AgCl, AgO_*x*_, Ag–S–O) with time. Finally, the absorbance decay and solution discoloration could be due, to some extent, to the formation of Ag^+^ ions, upon NP dissolution.^54,66^ Hence, the UV-vis spectra support the hypothesis of a change in the NPs chemical composition upon atmospheric exposure, thereby supporting the STEM-EDX observations.

To assess the interface between the NPs and the rapeseed oil, XPS measurements were performed on the pure oil and NP solutions. It should be noted that this method provides only a very limited probing depth^67^ of ∼4 nm for Ag,^68^ and ∼9 nm for organic compounds.^69^ The survey scans of pure oil samples and NP solutions showed the presence of only Ag, O and C (SI, Fig. S4). While Ag–AgCl NPs were present in the solutions, no Cl was detected in the probed volume which is probably due to a reorganization of the NPs and oil molecules under vacuum. Also, no S was detected. The overall quantification, based on the high-resolution spectra of the C 1s, O 1s and Ag 3d regions (SI, Fig. S5), revealed an increase in the O content upon atmospheric exposure from ∼8 at% in the asdep state to ∼13 at% at day 35, whereas the C content remained overall constant. The Ag content decreased from ∼5 at% in the asdep state to ∼1 at% at day 35. This drop was attributed to the formation of oxidation products with higher vapor pressure compared to the oil triglycerides, preferentially populating the surface under vacuum and thereby increasing the proportion of C and O present in the first nanometers below the liquid surface.

**Fig. 6:**
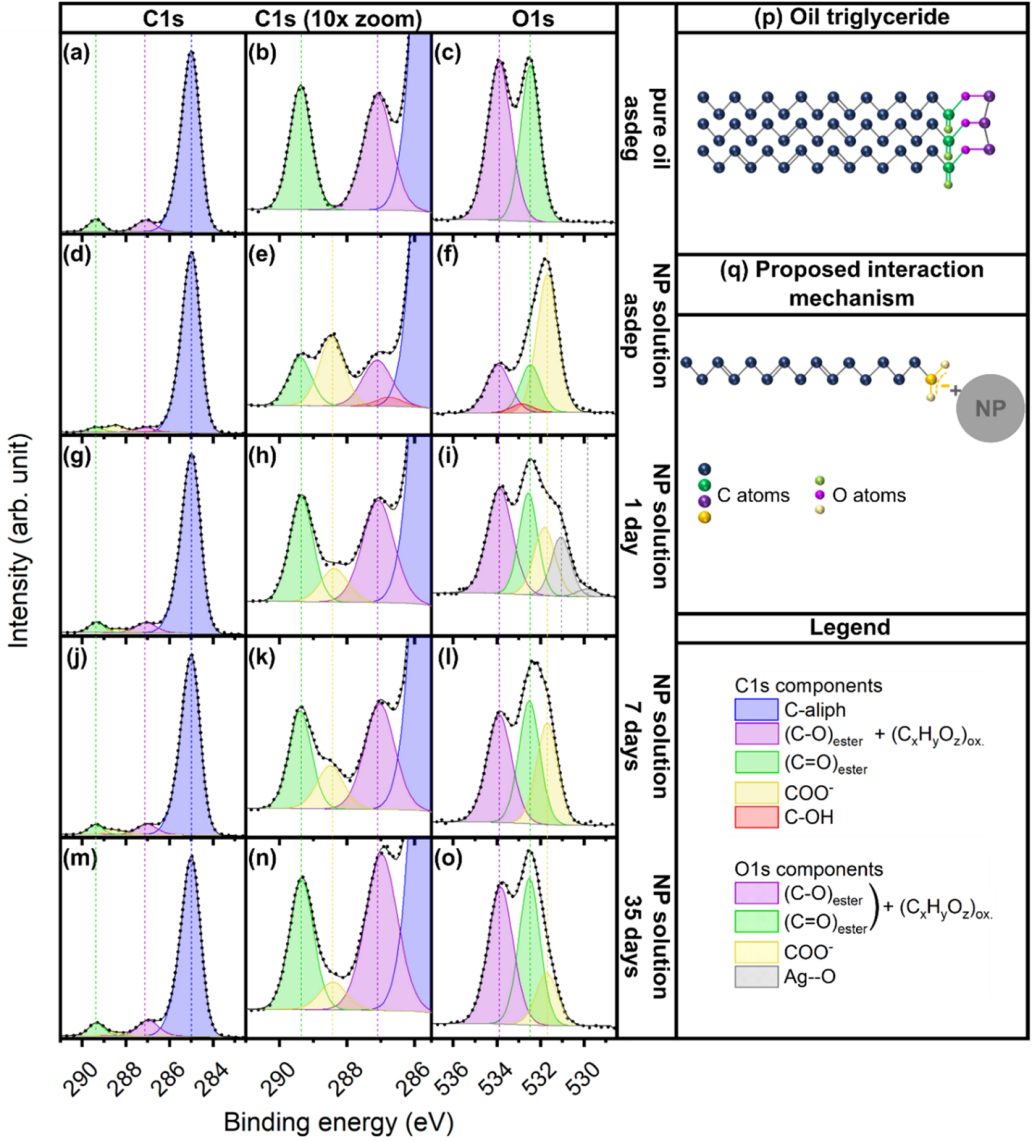
C 1s and O 1s XPS spectra of the pure oil ((a) to (c)) and the NP solutions in the asdep state ((d) to (f)), at day 1 ((g) to (i)), day 7 ((j) to (l)) and day 35 ((m) to (o)). The dotted line represents the measurement, the solid black line the fit. A schematic representation of the pure oil triglyceride is given in (p), along with the proposed interaction mechanism in (q). (C_*x*_H_*y*_O_*z*_)_ox_ refers to overlapping contributions from oil oxidation products.

The C 1s and O 1s signals of all samples shown in Fig. 6, were fitted using the strategy described in the SI. Starting with the pure oil (Fig. 6(a)–(c)), whose structure is shown in Fig. 6(p), the C 1s spectrum was fitted with 3 components (Fig. 6(a) and (b)). The main contribution at 285 eV arose from the aliphatic part of the molecule and corresponded to single and double C bonds of the backbone of the triglycerides, as well as C–H bonds. This contribution showed a slight asymmetry on the high binding energy (BE) side that can be attributed to the vibrational fine structure arising from the excitation of C–H stretch vibrations on the molecule backbone during photoionization.^36,38^ This asymmetry motivated the choice of mixed Gaussian–Lorentzian (70–30%) vibrational components for the fit of this peak. The signal from the last carbon atom on the C backbone before the ester group was also accounted for in this contribution, which will be referred to as C-aliph. The C atoms singly bonded to O in the glycerol part of the molecule gave rise to the component at ∼287 eV (C–O). Finally, the C 1s peak visible at ∼289.4 eV was attributed to the C atom in the ester group (–(C=O)–O–). This contribution will be referred to as C=O. These two last contributions have their counterparts on the oxygen signal (Fig. 6(c)), at ∼534 eV for the singly bonded O atom and ∼532.5 eV for the doubly bonded O atom of the ester group (–(C=O)–O–). In order to simplify the notation, these contributions will be noted C–O and C=O, respectively. This description of the C 1s signal in the pure oil allowed to reach proportions of 89, 5 and 6 at% for the C-aliph, C=O and C–O, respectively. Considering only the contributions involving O, their share is 44 and 56 at% for C=O and C–O, respectively. The C=O and C–O components on the O 1s signal were found in the same proportions, reflecting the consistency of the fitting. From the structure of the triglyceride (Fig. 6(p)), proportions of 90, 5 and 5 at% were expected on C 1s for C-aliph, C=O and C–O, respectively, and 50 at% each for C=O and C–O on O 1s. The small difference with the experimental result, leading to a ratio of C–O to C=O of 1.3, both on C 1s and O 1s, is probably due to shake-up structures of the C=O unit in the ester group, reducing the intensity of the C=O components on both C 1s and O 1s.^36^ The fitting results were therefore in good agreement with the expected composition of the oil. Furthermore, these results showed that the free fatty acids were not preferentially measured, since this would have led to a much lower proportion of the C–O component on the C 1s signal, suggesting that the free fatty acids did not preferentially populate the oil surface under vacuum. Fig. 6(d)–(o) shows the C 1s and O 1s signals of the NP solution upon atmospheric exposure, and their fits (see also the overlay of the spectra in Fig. S6 of the SI). At all atmospheric exposure times, two peaks on C 1s and O 1s are present at the positions of C–O and C=O detected in the pure oil. Additionally, a new component is clearly visible at ∼288.5 eV on C 1s and ∼531.8 eV on O 1s, which was attributed to a carboxylate group –((C=O)–O)^−^ interacting with the surface of the NPs, and will be referred to as COO^−^ in the following. Its position on both C 1s and O 1s is in good agreement with reported positions of C and O atoms in metal carboxylate groups.^70,71^ Especially in the asdep state, where its proportion is the largest, a good fit could only be reached with an approximately twice as large proportion of this new contribution on the O 1s signal compared to its proportion on the C 1s signal, considering components involving both C and O. Such proportions are expected for the COO^−^ group, involving two O atoms and one C atom, and were constrained for all fits at all atmospheric exposure times. Additionally, the full width at half maximum (FWHM) and position of the COO^−^ component were constrained for all fits, based on their values in the asdep state. Based on this attribution, the proposed interaction mechanism between oil components and NPs is shown schematically in Fig. 6(q).

Upon sputtering, the oil matrix did not seem to undergo significant changes (see Fig. 2), therefore the ratio of C–O to C=O is expected to be equal to that in the pure oil. In these conditions, the best fit is obtained upon addition of a weak component at ∼286.7 eV on C 1s, which can be attributed to a –C–OH unit, overlapping with the C–O component. Accordingly, a –C–OH unit of corresponding intensity was implemented on the O 1s signal, and overlaps with both C=O and C–O components. On day 1, the implementation of the COO^−^ component is not sufficient to fit the whole O 1s signal, which broadens on the high BE side. To account for this broadening, two further components are required, at ∼530 and ∼531 eV, and denoted Ag–O (1) and Ag–O (2). Such additional features are not detected on the C 1s signal, indicating that the corresponding chemical group does not involve C atoms. Furthermore, they are only present on day 1, suggesting their transitory nature. While signals in this BE range have been associated with O bulk species in Ag,^72,73^ or Ag surface oxidation,^73,74^ they most likely arise from adsorbed oxygen species at the NP surface^74–76^ and hydroxide groups (–OH).^77,78^ Regarding the components arising from the ester groups (C–O and C=O) on both O 1s and C 1s, their ratio (∼1.4) is close to that found in the pure oil, even without constraining their relative areas. This is in good agreement with the findings from the ATR-IR spectroscopy measurements, which only showed very limited oil oxidation at day 1. On days 7 and 35, however, the oil oxidation is clearly visible (Fig. 2). The formation of hydroperoxides, peroxides and epoxides upon oil oxidation^33^ is expected to yield a contribution on the C 1s and O 1s spectra at ∼286–288 and ∼533–534 eV, respectively.^36,79^ The strong overlap of these positions with the C–O signal from the ester group in the case of C 1s and with both the signals from C–O and C=O in the case of O 1s do not allow decoupling their share. The ratio of C–O to C=O on C 1s and O 1s is therefore no longer equal to that of the pure oil. This ratio might be further altered by the presence of signals from shorter chained secondary compounds, for instance C=O from ketones or aldehydes, which would give rise to peaks at ∼287.9 eV and ∼532.3 eV, on C 1s and O 1s, respectively.^36^ Therefore, the relative proportions of the C–O and C=O components were not constrained during the fit for days 7 and 35, and ratios of 1.5 and 1.7 were reached, respectively, for C 1s. For the O 1s signal, ratios of 1.1 and 1.2 were determined for days 7 and 35, respectively. These values suggest the presence of additional contributions, affecting differently the C 1s and O 1s spectra, and reflect the observed oil oxidation. Thus, the analysis of the C 1s and O 1s XPS spectra reveals that Ag interacts with the carboxylate groups of the fatty acids, and that this interaction does not undergo significant changes upon atmospheric exposure.

**Fig. 7:**
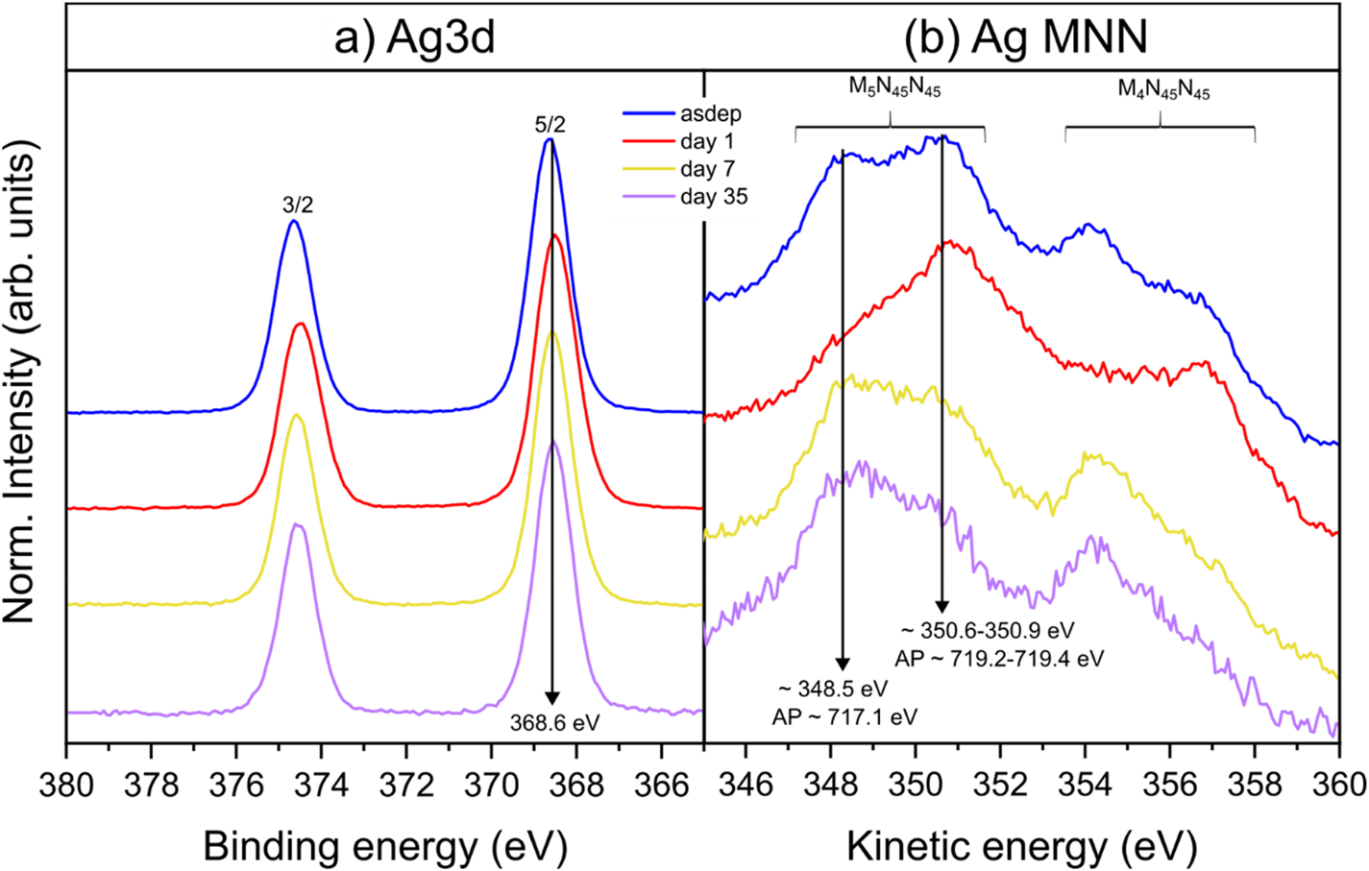
(a) Ag 3d spectra and (b) Ag Auger spectra (asdep to 35 days from top to bottom). The calculated Auger parameter (AP) for the main features in Ag M_5_N_45_N_45_ and Ag 3d 5/2 is indicated on the corresponding Auger peaks.

The Ag 3d and Auger signals, shown in Fig. 7(a) and (b), respectively, further support that the interaction between NP and oil takes place *via* the carboxylate groups. The absence of satellite peaks on the Ag 3d spectra, the symmetrical peak shape and the larger peak width, as compared to bulk Ag (SI, Fig. S7) suggest that most of the probed Ag atoms are not in a metallic state, regardless of the storage time. The Ag 3d components (5/2 and 3/2) are separated by 6 eV, with 5/2 located at 368.6 eV, *i.e.* 0.4 eV higher than the reported value for bulk Ag (368.2 eV).^80,81^ Both components appear to have shifted by −0.1 eV from day 1 on, compared to the asdep state. However, this shift is within the uncertainty on the peak positions; therefore, no significant changes were detected on the normalized peak upon atmospheric exposure. BE shifts are influenced by the chemical environment of the species investigated. In bulk materials, Ag oxides (stable Ag_2_O, AgO) and AgCl would give rise to a negative core-level BE shift.^80–84^ However, for supported and embedded metal NPs, size effects were reported to be responsible for peak broadening and core-level BE shifts, notably depending on the interaction between the NP and the surrounding material.^85,86^ For an interaction between a positively charged Ag NP surface and a carboxylate group (COO^−^), higher binding energies than for metallic Ag can be expected, as it is the case for Ag salts such as Ag acetate (CH_3_CO_2_Ag).^80,87^ As for the Auger spectrum of Ag seen in Fig. 7(b), its shape does not correspond to the Auger peak recorded for metallic Ag (SI, Fig. S7), but shows strong similarities with the Auger spectra reported for Ag in the ionic carbonate compound Ag_2_CO_3_.^88,89^ Similar to the carbonate, the carboxylate group of a fatty acid can interact with metal cations like Ag^+^ or positively charged metallic NPs,^90^ affecting hence the appearance of the Auger spectrum. Furthermore, the calculated values of the modified Auger parameters (AP), using the two main peaks detected in the Auger feature M_5_N_45_N_45_, and the core level Ag 3d 5/2, do not correspond to metallic Ag (717.1–719.3 eV *vs.* 720.2–720.5 eV, see Fig. S7(b) and (ref. 91)). The AP of 717.1 eV, however, is close to the range reported for Ag acetate (716.5–716.7 eV).^87^ Hence, the interaction, between Ag at the NP surface and carboxylate groups can explain the characteristics of the Ag 3d and Ag MNN peaks recorded. Finally, the shape of the Auger peak at day 1 strongly differed from the other days, which supports the observation of a transitory state on the O 1s signal on day 1.

Hence, the XPS analysis showed that a portion of Ag NPs interact with the fatty acids *via* their carboxylate group. Such interactions were also observed by Nguyen *et al.* for Au NPs produced by SoL using oleic acid and a mixture of oleic acid and oleylamine.^7^ This is also in line with the fact that fatty acids like oleic acid (the main component of rapeseed oil) are often used as capping agents to stabilize NPs produced by wet chemistry methods.^92,93^ However, Wender *et al.* proposed that Ag NPs produced by SoL interact with the olefinic bonds of unsaturated triglycerides of the host liquid, canola oil, whose composition is close to that of rapeseed oil.^5^ The findings presented herein suggest that this hypothesis does not capture the complete interaction mechanism, since the possible involvement of the carboxylic groups of the fatty acids was not considered.

Table 3 summarizes the proportions of the different contributions obtained for the pure oil and the NP solutions at all atmospheric exposure times. These proportions will be used to gain further insights in the interaction mechanism; nonetheless, they should be considered carefully due to the inhomogeneity of the system studied herein. The formation of carboxylate ions could take place either by simple deprotonation of the carboxylic group of the free fatty acids (FFAs) present in the oil or by breaking a triglyceride chain, thus releasing a fatty acid. The number of FFAs in the pure oil can be estimated based on its acid value (0.1 mg KOH per g). Despite the low acid number of the oil used in this investigation (refined oil), the FFAs still account for about 0.16% in number of all molecules in the oil. For 1 mL of oil, this corresponds to ∼10^18^ FFAs for ∼6 × 10^20^ triglycerides. Based on the median NP size determined in the asdep state, the Ag concentration in solution corresponds to ∼7 × 10^16^ NPs in 1 mL of oil. Therefore, the number of FFAs would be sufficient to interact with the NPs present in the solution, without the necessity to break triglyceride chains. However, on the C 1s signal, the proportion of C-aliph over the total C contribution slightly increases upon NP formation (see pure oil asdeg *vs.* asdep in Table 3). Two scenarios can explain this trend. On the one hand, chain splitting of a triglyceride would leave a C atom on the glycerol part of the triglyceride, no longer bound to an O atom, at the position of the chain splitting. This C atom then contributes to the C-aliph component. In the asdep state, part of these C atoms at the position of the chain splitting may form –C–OH bonds, explaining the –C–OH component included in the fit of the C 1s signal in the asdep state. This requires the presence of O atoms that were not present in the degassed pure oil. An incorporation of O atoms during the short sample handling prior to XPS measurement is plausible, with the increase in the ratio between O and C content in the asdep sample, compared to the pure oil, as seen from Table 3 and Fig. S5. Thermodynamically, the decomposition of triglycerides into FFAs and glycerol can take place under addition of water at high temperature (250 °C), but is not possible at room temperature.^94^ However, during the sputtering process, the liquid receives energy from the plasma.^3,95^ While the temperature of the liquid is expected to rise only by a few degrees over the deposition time,^3^ the incoming energy might be, locally and over the timescales of molecular vibrations, much higher than recorded through a temperature measurement. Furthermore, Ag NPs are commonly used for the catalysis of organic chemical reactions.^96^ Therefore, chain breaking of triglycerides might be promoted by the presence of NPs and incoming Ag atoms, and by the available energy from the process. On the other hand, the increase in the C-aliph component over the total C contribution upon NP formation might be due to the formation of AgCl. The Cl is present in the oil in the form of bound monochloropropane-1,2-diol (MCPD). Oil molecules bearing a Cl atom have a similar structure as a triglyceride or diglyceride, with a fatty acid chain replaced by a Cl atom. When leaving the molecule upon reaction with Ag, a C atom is left on the glycerol part that contributes to the C-aliph component. Part of these C atoms might in turn form –C–OH bonds upon atmospheric exposure.

**Table 3:** Summary of the XPS quantification of the pure oil and solutions

Proportions (at%)		Pure oil, asdeg	asdep	day 1	day 7	day 35
Contributions on C 1s	C-aliph	81.9	80.2	74.8	77.1	73.5
	C–O^a^	5.5	2.2	4.9	5.0	7.1
	C=O	4.4	1.7	3.5	3.4	4.2
	COO^−^		2.5	1.4	1.7	1.2
	C–OH		0.4			
**Total C**		**91.8**	**86.9**	**84.6**	**87.2**	**86.1**
Contributions on O 1s	C–O^a^	4.6	1.9	3.7	3.8	5.6
	C=O^a^	3.6	1.4	2.7	3.4	4.8
	COO^−^		4.6	2.0	3.1	2.1
	C–OH		0.3			
	O–Ag (1)			1.6		
	O–Ag (2)			0.3		
**Total O**		**8.2**	**8.3**	**10.3**	**10.3**	**12.5**
**Total Ag**		**0**	**4.9**	**5.1**	**2.4**	**1.4**

a In case of oil oxidation, component also includes overlapping contributions from further groups that cannot be resolved separately.

Considering the proportions of detected Ag and Ag-involving contributions on O 1s, Table 3 shows an overall good correlation between the Ag content and the intensity of the COO^−^ signals on O 1s, except on day 1. However, on day 1, the Ag atoms are not only interacting with the carboxylate groups, but also with the adsorbed oxygen species (Ag–O (1) and (2)). The total contribution of oxygen species interacting with Ag atoms (3.9 at% at day 1) follows the same trend as the Ag content over time. Furthermore, this correlation, including the detection of adsorbed oxygen species, suggests that the Ag detected is part of a NP and not a single Ag^+^ ion, despite the fact that the formation of Ag^+^ ions in solution under oxidative conditions cannot be excluded.^54,66^ Moreover, since the Ag atoms detected are mostly present in a non-metallic state and do not seem to be part of a Ag oxide phase, they were most probably mostly located at the very surface of Ag NPs. The proportions reported in Table 3 also reflect the progressive oxidation of the oil molecules that was evidenced by FTIR (Fig. 2). After deposition, the decrease in proportion of aliphatic component on the C 1s signal with increasing atmospheric exposure time can be associated to the formation of oxidation products, with, for instance, hydroperoxide groups attaching to the carbon backbone of the triglycerides.^33^ The components on the O 1s XPS signal, attributed to adsorbed oxygen-containing species on the NP surface (Ag–O (1) and (2)) are probably the first signs of the oil oxidation process, catalysed by the Ag NPs.^97^ Finally, the overall decrease in detected Ag over time can also be explained by this change in oil chemistry, especially the presence of shorter chained oxidation products, which tend to move to the sample surface due to their lower vapor pressure and/or their lower density, as compared to the triglycerides. When increasing the time of exposure to vacuum prior to measurement up to several days, the detected Ag content and proportion of COO^−^ component on C 1s and O 1s tend to increase again, thereby supporting this explanation.

## 4. Conclusion

NP solutions were produced by magnetron sputtering of a Ag target onto refined rapeseed oil and exposed to the atmosphere for up to 35 days. The as-deposited solutions exhibited a dark brown colour, originating from the LSPR of Ag NPs, indicating that the oil sufficiently stabilizes the NPs to prevent strong aggregation. The LSPR was located at ∼435 nm, as revealed by UV-vis measurements. The log-normal size distribution was characterized by a median NP diameter of 1.4 (−0.3/+0.4) nm, with only ∼10% of all NPs > 2 nm. Besides metallic Ag NPs, Ag–Cl compounds were detected by TEM-EDX and electron diffraction. The Cl originated from contamination present in the oil and led to the formation of Ag–AgCl Janus-type NPs already in the as-deposited state.

Within 35 days of atmospheric exposure, the solution colour gradually evolved to light orange, suggesting structural and/or chemical changes of the NPs and/or oil matrix. A size increase was detected upon atmospheric exposure, with almost 20% more NPs > 2 nm after 35 days, indicating that the particles continue to grow in the solution after sputtering. While these changes contributed to the modifications of the UV-vis absorbance spectra with time, they could not explain, alone, the strong redshift of the LSPR of ∼15 nm and an absorbance decay by ∼95% after 35 days. These temporal evolutions were attributed to the continued formation of Ag–AgCl Janus-type NPs with time, upon exposure of the NPs to the Cl-containing oil, and to the formation of further Ag compounds, such as Ag–S–O. While not detected, the formation of Ag (sub)oxides at the surface of the NPs cannot be excluded and would also contribute to the observed changes.

IR spectroscopy measurements showed that the oil matrix underwent oxidation upon atmospheric exposure, especially with the formation of hydroperoxides. The changes in IR signal intensified faster for the NP solutions than for the pure oil, demonstrating that oil oxidation is accelerated by the presence of Ag NPs. Finally, the comparison of XPS spectra of the pure oil with as-synthesized and aged NP solutions revealed the presence of carboxylate groups (COO^−^) interacting with Ag. Such an interaction implies the opening of triglyceride chains and/or the deprotonation of already present free fatty acids. Along with the fingerprints of this interaction, observed at all atmospheric exposure times, the contributions from chemical groups of the oil molecules were analyzed and the changes with time in their relative intensities reflected the oxidation of the oil matrix.

Not only do these results shed light on the interaction between metal NPs and oil molecules and on solution stability, they also show the mutual influence of NP and liquid on each other. In particular, trace contaminants in the host liquid can play a crucial role for the chemistry of the produced NPs, while NPs can accelerate the oxidation of the host liquid. Finally, this study exemplifies the importance of a comprehensive characterization of the NP-liquid system, allowing for knowledge-based material choice and experimental design.

## Author contributions

Heloise Lasfargues: conceptualization, methodology, validation, investigation, formal analysis, data curation, writing – original draft, writing – review & editing, visualization, project administration. Devi Janani Ramesh: methodology, validation, investigation, formal analysis, data curation, writing review & editing. Lilli Charlotte Freymann: methodology, validation, investigation, formal analysis, data curation. Jochen M. Schneider: writing – review & editing, supervision, resources. Clio Azina: conceptualization, writing – review & editing, supervision, funding acquisition, project administration.

## Conflicts of interest

The authors declare that they have no known competing financial interests or personal relationships that could have appeared to influence the work reported in this paper.

## Supplementary Material

NA-OLF-D5NA01153A-s001

## Data Availability

The data supporting this study are available within the article and the supplementary information (SI). Supplementary information is available. See DOI: https://doi.org/10.1039/d5na01153a.
