# Supplementary material for: Combined spectroscopic and microscopic investigation of stability upon atmospheric exposure of Ag nanoparticle solutions produced by sputtering onto rapeseed oil
Source: Nanoscale Adv. 2026 Mar 19;8(8):2628–44. doi: 10.1039/d5na01153a (PMC13011856; doi:10.1039/d5na01153a)
Supplement: NA-008-D5NA01153A-s001 [file NA-008-D5NA01153A-s001.pdf]

Supplementary Information (SI) for Nanoscale Advances.
This journal is © The Royal Society of Chemistry 2026

Combined spectroscopic and microscopic investigation of stability upon atmospheric exposure of Ag nanoparticle solutions produced by sputtering onto rapeseed oil

Héloïse Lasfargues[1*], Devi Janani Ramesh[1], Lilli Charlotte Freymann[1], Jochen M. Schneider[1] and Clio Azina[1,a]

[1] Materials Chemistry, RWTH Aachen University, Kopernikusstraße 10, 52074 Aachen, Germany

[*] Corresponding author; email: lasfargues@mch.rwth-aachen.de

[a] Present address: Laboratoire des Composites Thermostructuraux, CNRS, Univ. Bordeaux, Safran Ceramics, CEA, 3, allée de La Boétie, 33600, Pessac, France

Supplementary information

Sputtering onto rapeseed oil

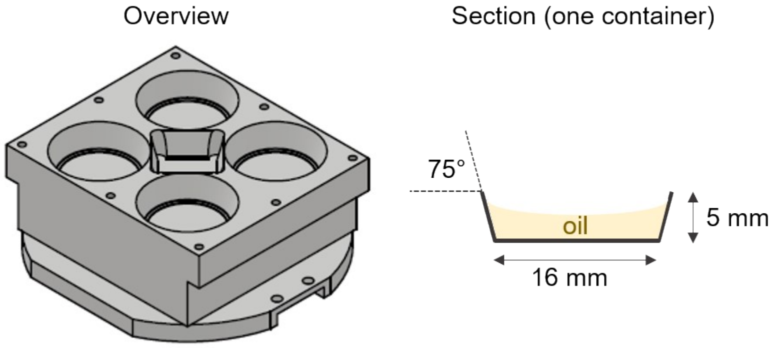

Figure S1: Liquid holder used for SoL

STEM – (HR)TEM – SAED

Table S1: Imaging and evaluation choices for particle statistics.

Magnification / Field of view (µm²)	Minimal total number of images analyzed	Size range analyzed (nm)	Average number of particles evaluated	Pixel size (nm)
x50k / 11.43	7	>20	134	2.7
x150k / 1.27	11	>10	92	0.90
x350k / 0.23	11	5-10	50	0.39
x500k / 0.11	15	0-7	3480	0.27

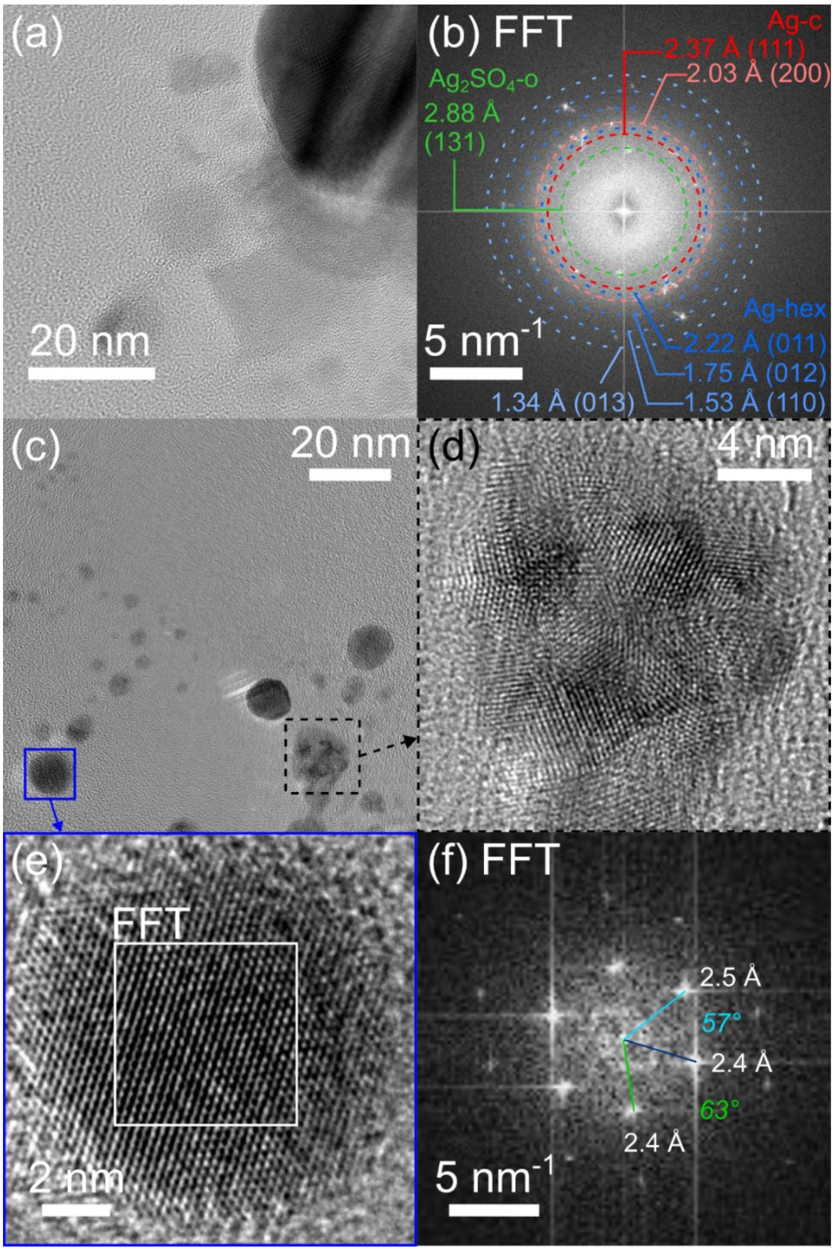

Figure S2: (a and c) HRTEM images of selected regions of the asdep sample. (b) FFT pattern corresponding to (a). (d) Enlargement from (c) showing a polycrystalline NP. (e) Enlargement from (c) showing a twinned cuboctahedral NP [1], with corresponding FFT pattern (f). “c” refers to cubic, “o” to orthorhombic and “hex” to hexagonal.

[1] H. Hofmeister et al., “Shape and Internal Structure of Silver Nanoparticles Embedded in Glass,” *Journal of Materials Research* 20, no. 6 (2005): 1551–62, https://doi.org/10.1557/JMR.2005.0197.

XPS – Methodology and analysis

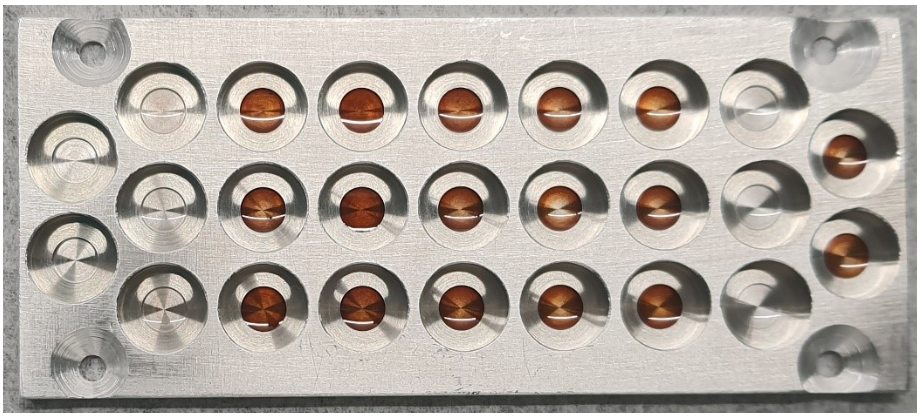

Figure S3: Sample holder for XPS measurements, filled with pure oil and NP solution (orange color).

Measurement and fitting strategy adopted for XPS analysis:

- The sample holder design and measurement parameters were chosen to minimize exposure of the sample to X-rays, while ensuring sufficient signal-to-noise ratio and resolution. The design provided sufficient measurement areas of non-exposed sample to perform all scans (statistics for global quantification, signal averaging for Ag MNN peak).
- The measurements were done on two samples produced and handled in the same conditions. The spectra showed very similar behavior with atmospheric exposure.
- The fitting and quantification were performed with both Tougaard and Shirley backgrounds for all core levels. The same trends were obtained, with only minimal differences in the proportions measured. Since most of the measured elements are organics, the Tougaard background was chosen.

- The peak models obtained for the pure oil signals (as-degassed) were used as a basis to fit the signals from the NP solutions.
- In particular, the peak positions and FWHM from C═O and C─O components were constrained within 0.1 eV from their position on the pure oil. For the COO^- component, position and FWHM from the asdep sample were used as basis (constraint within 0.1 eV). For other components implemented, the FWHM was kept in similar ranges (1-1.2 eV).
- No significant changes on the C1s and O1s spectra and proportions of C and O of the pure oil upon atmospheric exposure were observed.

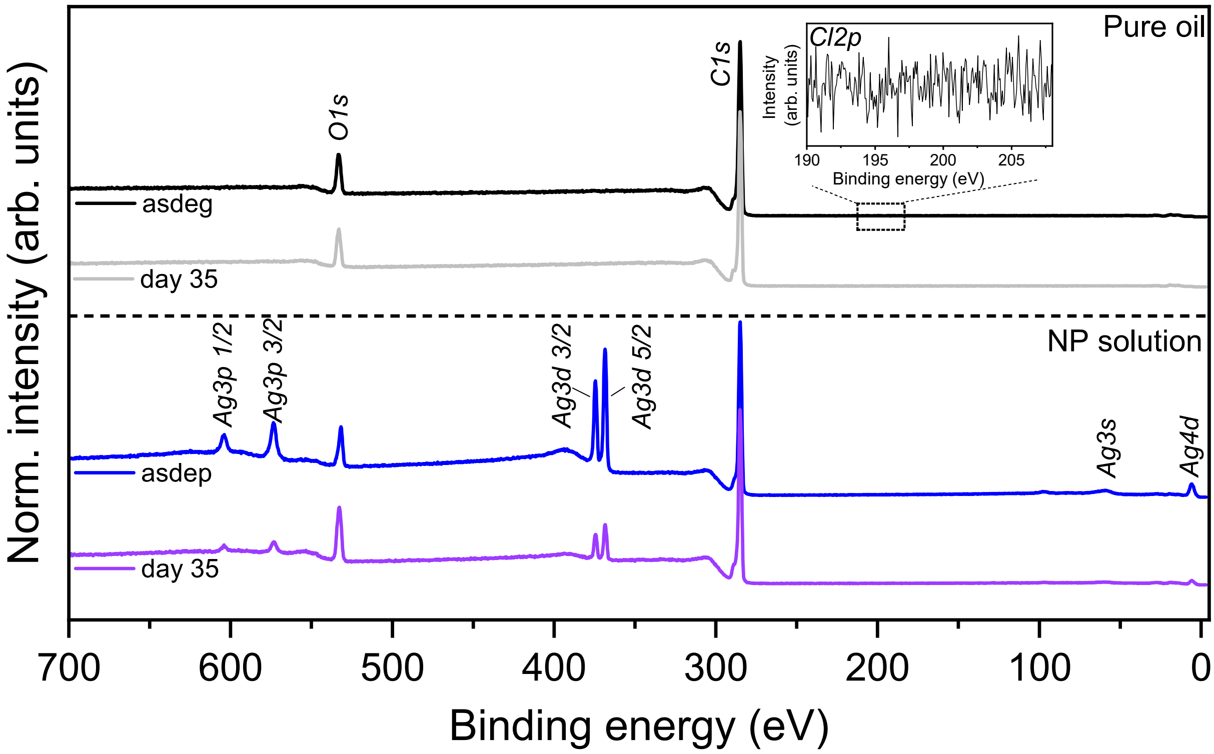

Figure S4: Survey spectra of the pure oil (top) and NP solution (bottom), in the as-degassed and as-deposited state, respectively and after 35 days atmospheric exposure. The inset shows a high-resolution scan of the Cl2p region of the pure oil, showing that no Cl is detected by XPS. Also, no S was detected (S2p in the range 160-170 eV).

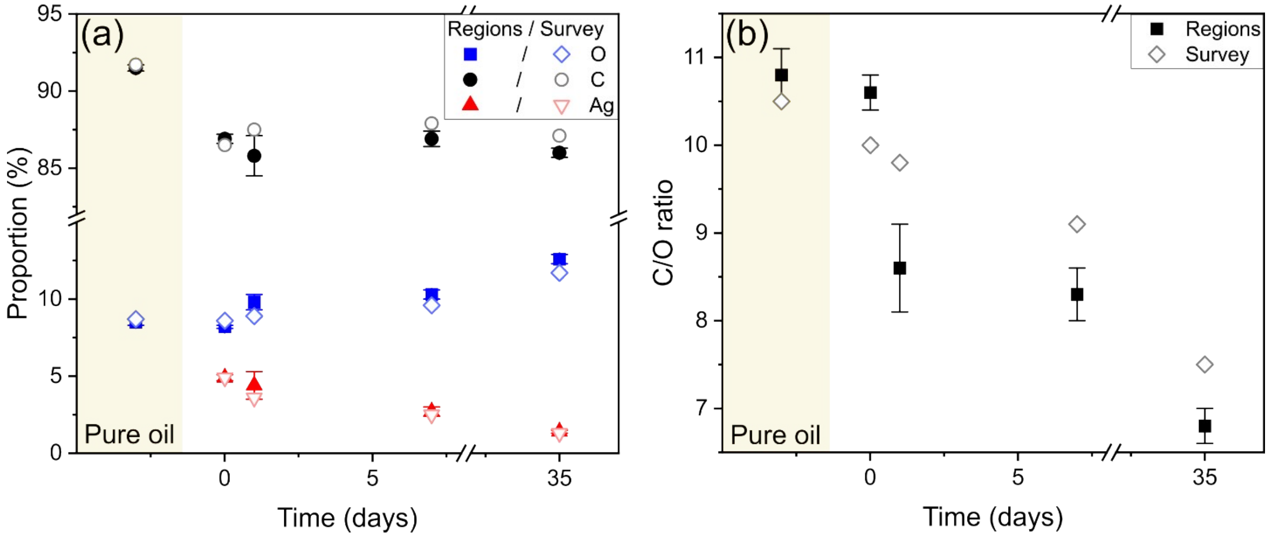

Figure S5: Quantification based on XPS high-resolution spectra (“Regions”) and on survey scan, using the O1s, C1s and Ag3d peaks. Evolution of (a) proportions of C, O and Ag and (b) C/O ratio, in the probed volume with atmospheric exposure time. The quantification was carried out for each position, on which O1s, C1s and Ag3d regions were acquired. The values and error bars reported are the average and standard deviation of the results obtained, respectively.

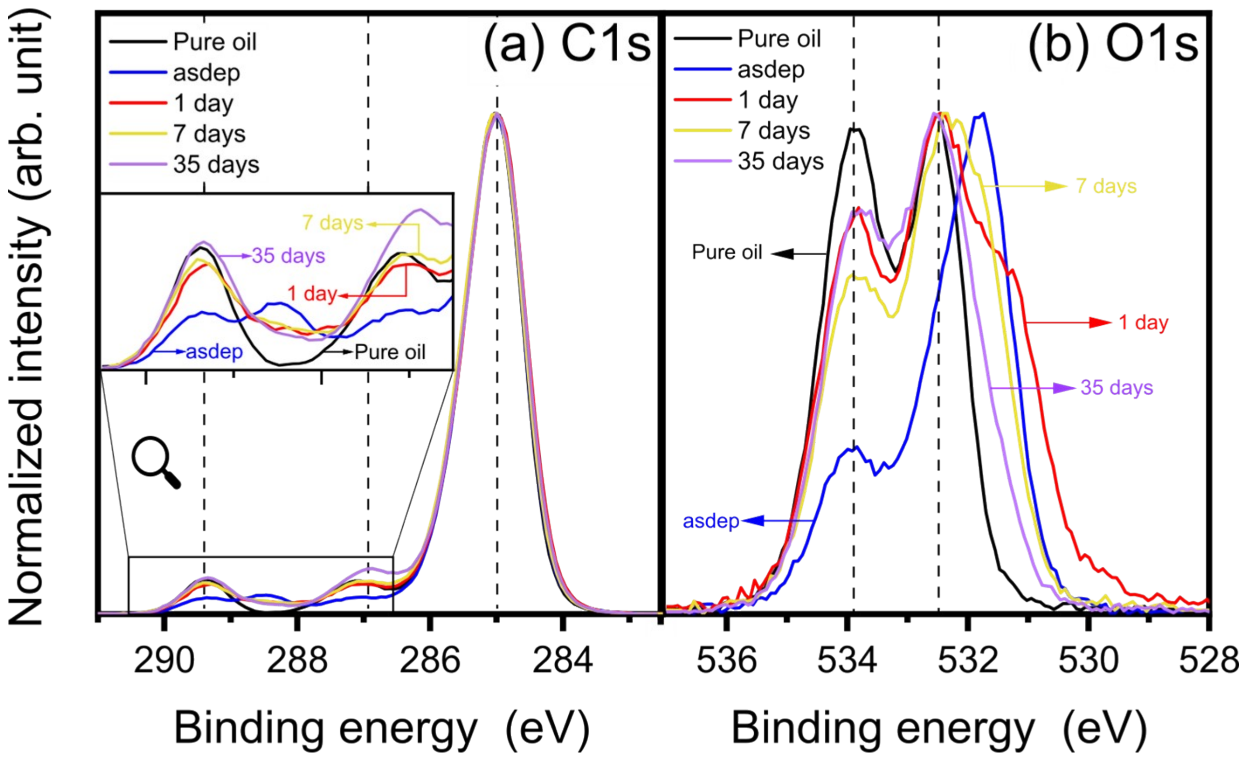

Figure S6: High-resolution C1s (a) and O1s (b) spectra of the NP solutions at different atmospheric exposure times. The inset in (a) shows the zoomed in spectra for binding energies between 286.5 and 290.5 eV. The intensities were normalized at the signal maximum for better visualization of the changes compared to the pure oil and upon atmospheric exposure.

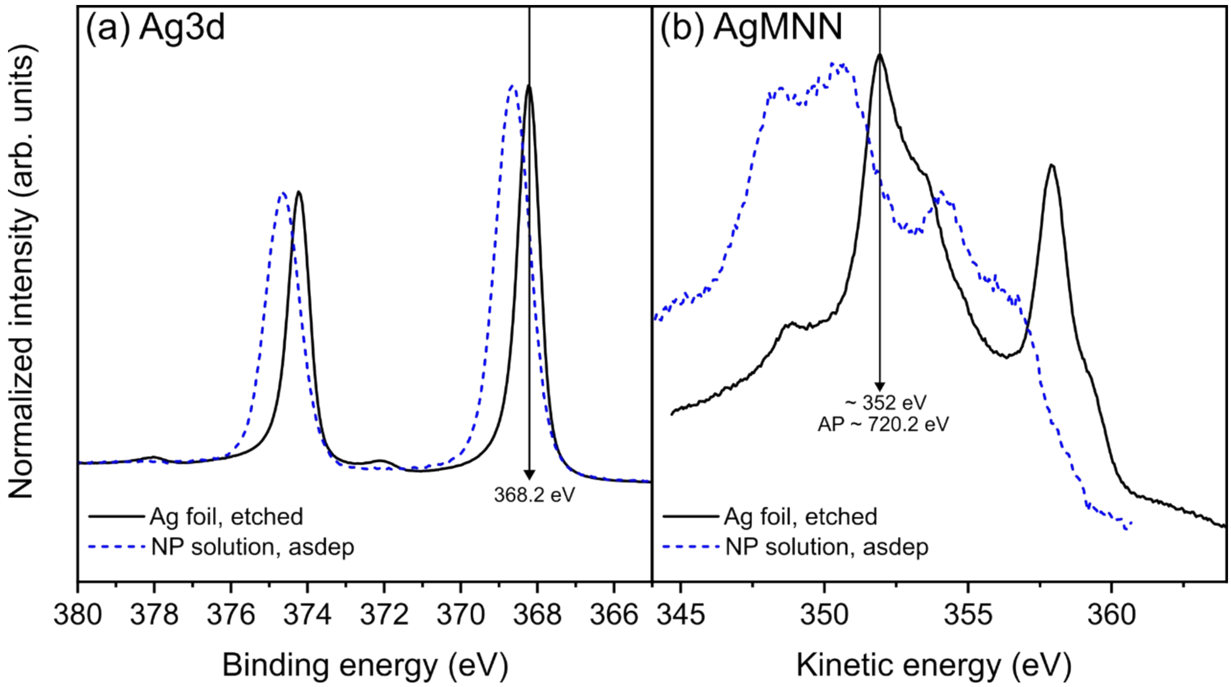

Figure S7: Ag3d (a) and AgMNN (b) signal of an etched Ag foil and comparison with the signal from asdep NP solution. The spectra of the etched Ag foil were acquired with a pass energy of 40 eV, as was used for the NP solutions.
